# Effects of cadmium at sub-lethal concentration on growth and biochemical parameters in rainbow trout (*Oncorhynchus mykiss*)

**DOI:** 10.1186/2046-0481-66-11

**Published:** 2013-06-20

**Authors:** M Saeed Heydarnejad, Mozhdeh Khosravian-Hemamai, Amin Nematollahi

**Affiliations:** 1 Fish and Fisheries Department, Faculty of Science, Shahrekord University, PO B 115, Shahrekord, Iran; 2 Faculty of Veterinary, Shahrekord University, POB 115, Shahrekord 88186, Iran

**Keywords:** Rainbow trout, Heavy metal, Cadmium, Growth

## Abstract

Cadmium (Cd), as one of heavy metals and an environmental stressor, may alter many physiological processes like growth and serum parameters in fish. The main objective of this study was to determine the effects of cadmium at sub-lethal concentrations (1 and 3 μg/l) on growth and serum biochemical parameters including enzymes, i.e. alkaline phosphatase (ALP), aspartate aminotransferase (AST) and alanine aminotransferase (ALT), glucose, triglyceride, cholesterol and total protein in rainbow trout (*Oncorhynchus mykiss*). Trout were exposed to cadmium, and, at intervals of 1, 15, and 30 days, selected parameters were evaluated. Condition Factor (K), Specific Growth Rate (SGR) and Body Weight Gain (BWG) consistently decreased, while Food Conversion Ratio (FCR) increased at the end of experiment. Glucose was elevated in trout exposed to both Cd concentrations at day 15 and then returned to levels comparable to control fish. Triglyceride and cholesterol decreased transiently at day 15 and then increased at day 30. Total protein, AST, ALT and ALP increased linearly by time and Cd concentration. This investigation suggests that growth and serum biochemical parameters could be used as important and sensitive biomarkers in ecotoxicological studies concerning the effects of metal contamination and fish health.

## Background

The problem of heavy metals accumulation in aquatic organisms including fish needs continuous monitoring due to biomagnifying potential of toxic metals in human food chain [1-6]. Due to the damage caused to the aquatic life, the pollution of freshwaters with a wide range of metals has become a matter of great concern over the last few decades [7]. Heavy metals may enter into aquatic ecosystems and induce stress symptoms in fish. Some metals are essential since they play an important role in biological systems, while some others are nonessential metals as they have no known role in biological systems [8,9]. One of the most important characteristics of toxic pollutants such as metals is that they can be accumulated in organs of the organisms [10]. Fish have been largely used in the evaluation of the quality of aquatic systems. These organisms are often at the top of the aquatic food chain and may concentrate large amount of metals from the surrounding waters [11]. The accumulation of metals in aquatic systems suggests that fish may serve as a useful indicator of contaminating metals in aquatic systems, since they respond with great sensitivity to changes in the aquatic environment [12,13].

Cadmium is a common environmental pollutant [14]. Cadmium is one of the most toxic heavy metals with a wide distribution. Estimation of responses to heavy metals may provide sensitive indicators on which to predict the effects of heavy-metal pollution on fish populations [15]. Exposure of fish to heavy metals could have widespread detrimental effects on their health [15]. Some of the physiological effects of chronic exposure to waterborne cadmium at sub-lethal levels are manifested in the form of disturbances in respiration [16,17], reduction in growth [18], disruption in whole-body or plasma ion regulation [19,20], changes in hematology [21-24], enzyme activity [24,25] and other blood parameters, such as glucose, total protein, triglyceride and cortisol that reveal the stress response in fish [26,27].

Cadmium concentration at sub-lethal levels have been found to decrease in growth in juvenile and adult rainbow trout (*Oncorhynchus mykiss*) [18], as well as to mortality and reduced growth in juvenile bull trout (*Salvelinus confluentus*) [28] and guppy (*Poecilia reticulate*) [29]. Serum enzymes such as alkaline phosphatase (ALP), alanine transaminase (ALT) and aspartate transaminase (AST) are important serum markers to study the health of animal species in question. The main objective of this study was to determine the effects of cadmium at sub-lethal concentrations (1 and 3 μg/l) on growth and serum biochemical parameters including enzymes (ALP, AST and ALT), glucose, triglyceride, cholesterol and total protein in rainbow trout (*Oncorhynchus mykiss*). Generally, an exposure concentration which is lower than LC_50_ is considered as sub-lethal. The preliminary experiment showed that 72 h-LC_50_ of cadmium was 9 μg/L. Therefore in this study, the sub-lethal doses of cadmium (1 and 3 μg/l) were determined according to 1/9th and 1/3th of the 72 h-LC_50_.

## Methods

### Fish holding conditions and acclimation

Rainbow trout (*Oncorhynchus mykiss*), matched for size (19.5±0.4 g; 12.5±0.3 cm), were transferred to the laboratory. They were kept in continuously aerated tanks (500 l) under a natural photoperiod (12 h light-12 h dark). Physicochemical characteristics of experimental water were as follows: temperature 9±1°C, pH 7.8±0.2, dissolved oxygen concentration, 7.8-8.3 mg/l, total hardness as CaCO3 104.2 mg/l, total alkalinity as CaCO3 78.8 mg/l and total dissolved solids 173 mg/l (Table 1). Temperature, DO (Dissolved Oxygen), TDS (total dissolved solids) and pH were monitored daily in all tanks. Hardness and alkalinity were measured four times per week in a subset of tanks by titrimetric methods. During an acclimation period of 2 weeks, the fish were hand-fed twice twice daily at random times with commercial dry pellets. The feed was comprised of 40.69±0.3% crude protein, 53.7±0.18% crude lipid, 25.59±0.11% ash and 11.13±0.5% moisture. Fish were fed at a feeding rate of 2% of body weight per day for 30 days. Any fish that showed abnormal behavior were removed immediately from the tanks.

**Table 1:** Mean (±S.D) water quality parameters, cations, anions, and background metals in acclimation

**Parameter**	**Acclimation water**
Temperature (°C)	9±1°C
pH	7.8±0.2
Dissolved oxygen (mg l^-1^)	7.8-8.3
Total Hardness (mg l^-1^ as CaCO3)	104.2
Total alkalinity(mg l^-1^ as CaCO3)	78.8
Total dissolved solids(mg l^-1^)	173
Sodium (mg l^-1^)	5.0
Calcium (mg l^-1^)	31.0
Potassium (mg l^-1^)	0.5
Magnesium (mg l^-1^)	6.4
Cl^-^ (mg l^-1^)	11.3
NH_3_(mg l^-1^)	0.05
SO_4_^-2^ (mg l^-1^)	15.0
PO_4_^-3^ (mg l^-1^)	0.05
Copper(μg l^-1^)	0.63
Cadmium (μg l^-1^)	0.57
Zinc (μg l^-1^)	91.37

The ethical approval for the study was obtained from Shahrekord University (SKU) Ethics Committee on 12th May 2008 (Ref: SKU-24-02-1387).

### Exposure system

Active groups of 20 fish were randomly transferred to 160l polyethylene exposure tanks with continuous aeration. The fish were exposed to: (i) control: nominally zero cadmium [actual measured ‘in-tank’ value: 0.07 μg/l], (ii) low cadmium [1 μg/l] and (iii) high cadmium [3 μg/l] for 1, 15 and 30 days. Cadmium added as CdCl_2_.H_2_O (Merck, Germany), each with three replicates. The water was changed every 2 days to minimize metal loss and maintain the concentration of metal. The water quality parameters mentioned were assessed at collection days during the experimental period. Growth performance was calculated as follows:

Condition Factor (CF) = 100 Weight (g)*/*Length^3^ (cm)

Specific Growth Rate (as percentage of body weight gain per day) = 100 × [ln final weight (g) - ln initial weight (g)] /time (days)

Feed conversion ratio (FCR) = feed intake*/*weight gain

Weight Gain Percentage = [final weight (g) - initial weight (g)] / initial weight (g)

### Sampling and biochemical processing

The fish were fasting for a 24-h period before sampling. Four fish were removed from each tank on days 1, 15 and 30 and anesthetized with clove oil (25 mg/l). Prior to and at the end of the experiment the weight and length of individual fish were measured. Fish blood samples were collected with a hypodermic syringe from the caudal vein. Blood samples were immediately kept in a refrigerator for 4 h. Serum was separated from cells by centrifugation of whole blood (10 min, 4000 g, 4°C) and stored at −48°C until the experimental assays.

The levels of ALT, AST, ALP with concentrations of glucose, triglyceride, cholesterol and total protein in the serum were measured using an Hitachi 911 biochemical analyzer (Japan).

### Statistical analysis

Initially the raw data were checked for normality of distribution by Kolmogorov- Smirnov tests. All values were expressed as means ± standard error of means (SEM). The analysis of differences between control and different sampling times in each exposure group and growth parameters was tested by one-way analysis of variance (ANOVA). The post hoc Duncan’s multiple range test were used among treatment means with SPSS 14. Significance was determined at P< 0.05.

## Results

### Growth

Fish exposed to higher Cd concentrations grew slower than fish exposed to the control and lower Cd concentrations (Table 2). This shows that the growth of test fish was dose-dependent. Significant (*P* < 0.05) differences were observed in the total weight gain and SGR of fish reared in water containing different concentrations of Cd as compared with the control. Weight gain and SGR decreased linearly with the increase of cadmium level in the water. The body condition factor of fish reared in water containing different concentration of Cd also decreased significantly (*P* < 0.05) as compared with the control. Exposure of the fish to different cadmium concentrations in water reduced feed consumption – a direct correlation with the levels of Cd concentration in the water. The FCR values increased with the level of cadmium concentration in water, indicating poor utilization of food in the fish. Results on the growth performance of *O*. *mykiss* subjected to different sub-lethal concentrations of cadmium are presented in Table 2.

**Table 2:** **Growth performance of rainbow trout ( *Oncorhynchus mykiss *) in control, low exposed (1μg/l) and high exposed (3 μg/l) cadmium over the experimental period of 30 days**

**Parameters/groups**	**Control**	**LowCd**	**HighCd**
**MWi**	19.51±0.5^a^	19.30±0.26^a^	19. 6±0.25^a^
**MWf**	48.39±1.03^a^	41.24±0.41^b^	31.71±0.55^c^
**MBLi**	12.3±0.32^a^	12.4±0.25^a^	12.65±0.10^a^
**MBLf**	15.96±0.12^a^	15.30±0.15^b^	14.30±0.15^c^
**CFi**	1.047±0.01^a^	1.012±0.01^a^	0.968±0.01^b^
**CFf**	1.190±0.01^a^	1.151±0.03^b^	1.085±0.01^b^
**SGR**	3.03±0.03^a^	2.53±0.2^b^	1.606±0.03^c^
**BWG**	148.153±10.85^a^	113.678±8.65^b^	61.88±6.5^c^
**FCR**	2.808±0.33^a^	3.156±0.56^b^	4.968±0.63^c^

*MWi:* Initial mean weight (g); *MWf:* Final mean weight (g); *MBLi:* Initial mean body length (cm); *MBLf:* Final mean body length (cm); *CFi:* Initial condition factor, *CFf:* Final conditionfactor; *SGR:* Specific growth rate; *FCR:* Feed conversion ratio. *BWG:* Body weight gain. Significant differences between values in columns are indicated with letters (*P <*0*.*05); values in columns with the same letters are not significantly different. Significant differences between values in columns are indicated with letters (*P <*0*.*05); values in columns with the same letters are not significantly different.

### Serum enzymes

**Figure 1:**
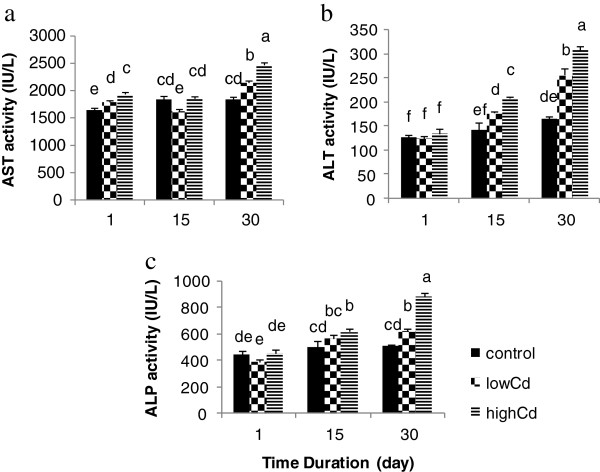
**Effects of different sub-lethal cadmium concentrations on serum enzymes activities. a**) Serum aspartate transaminase (AST), **b**) Serum alanine transaminase (ALT), **c**) Serum alkaline phosphatase (ALP) in *Oncorhynchus mykiss* exposed to cadmium. After 30 days, the AST and particularly the ALT levels increased and exhibited a linera pattern. The level of the ALP also showed a significant increase compared to the control group, when the fish were exposed to the both concentrations of cadmium. Data are expressed as mean±standard error (SE). Means with different letters are significantly different from each other (*P* < 0.05). Values with the same letters are not significantly different.

Both aspartate transaminase (AST) and alanine transaminase (ALT) activities exhibited a linear pattern and increased after 30 days (Figure 1). This increase was more remarkable in ALT activity for fish exposed to 3 μg/l Cd so that after 30 days a 128% increase was observed. Following by a transient reduction, AST activity also increased after 30 days. Similarity, this elevation was greater in high concentration of Cd (3 μg/l). The level of alkaline phosphatase (ALP) also exhibited remarkable increases in level from a mean control level, when the fish were exposed to the both sub-lethal concentrations of cadmium. This trend continued over time and with increasing Cd levels.

### Serum biochemical parameters

**Figure 2:**
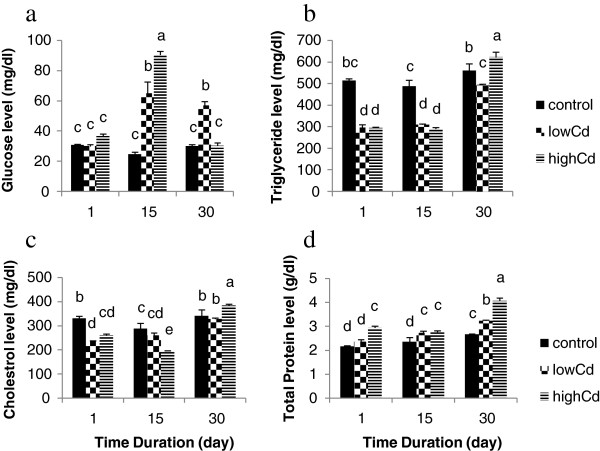
**Effects of different sub-lethal cadmium concentrations on biochemical parameters in *Oncorhynchus mykiss*. a**) Serum glucose level; after 15 days, the concentration of glucose increased but this elevation was transient so that after 30 days, the level of glucose returned to the same level in the control group, **b**) Serum triglyceride level; there was a decrease in the level of triglyceride for the first and 15^th^ days but it followed by a rapid elevation in 30 days, **c**) Serum cholesterol level; a significant reduction was observed after first and 15^th^ days, **d**) Serum total protein level; there was a significant increase after 30 days. Data are expressed as mean±standard error (SE). Means with different letters are significantly different from each other (*P* < 0.05). Values with the same letters are not significantly different.

After 15 days in both Cd- exposed groups’, the concentration of glucose increased and reached to 90.33±2.4 mg/dl compared to 30.67±0.6 mg/dl (control). This elevation in the glucose was transient and returned to the same level in the control group within 30 days (Figure 2a). After first and 15^th^ days of exposure to different concentrations of Cd, the level of triglyceride decreased to its minimum value and reached to 289.3±6.6 mg/dl (high Cd-exposed group) compared to 514±8.7 mg/dl (control group). These reductions were then followed by a rapid elevation in 30 days so that the level of triglyceride in serum in both Cd concentrations returned to the same level in the control group (Figure 2b).

Serum cholesterol showed a significant reduction after first and 15^th^ days in both Cd exposed groups and returned to the same level in the control group. In the first 15days, the cholesterol level decreased rapidly and reached to 260±10.3 mg/dl (low-dose) and196.6±0.8 mg/dl (high-dose) compared to 331±8.5 mg/dl in control group but this reduction was transient (Figure 2c).

Total protein in serum exhibited a linear pattern and increased after 30 days. This elevation was not significant in 15 days but increased rapidly to 3.2±0.08 g/dl (low-dose) and 4.06±0.1 g/dl (high-dose) compared to 2.6±0.03 g/dl in control group after 30 days (Figure 2d and Table 3).

**Table 3:** **Biochemical effects of cadmium in low exposed (1μg/l) and high exposed (3 μg/l) rainbow trout ( *Oncorhynchus mykiss *) in comparison with control over the experimental period of 1, 15 and 30 days**

**Parameters/days/groups**		**Control**	**LowCd**	**HighCd**
**Glucose(mg/dl)**	1	30.6±0.6	30±1.1	36.6±1.4
	15	24.6±1.4	65±7.5	90.3±2.4
	30	30±1.1	57±2.5	30.3±1.8
**Triglyceride(mg/dl)**	1	514±8.7	296.6±13.3	294.6±4.3
	15	486.6±29.6	309.3±3.7	289.3±6.6
	30	560.6±31.3	494.3±4.1	622±24.4
**Cholesterol(mg/dl)**	1	331±8.5	235.3±5.1	260.6±5.4
	15	287.6±23	260±10.3	196.6±1.8
	30	340.3±21.3	327.3±5.9	383±6.2
**Total Protein(g/dl)**	1	2.1±0.3	2.3±0.1	2.9±0.1
	15	2.3±0.1	2.7±0.1	2.7±0.1
	30	2.6±0.1	3.2±0.1	4.2±0.1
**AST(IU/L)**	1	1645.3±31.4	1789.6±21	1929.3±42.5
	15	1836.3±64	1622.3±39.2	1854.3±32.2
	30	18.33±51.3	2143.6±36.5	2468.3±40
**ALT(IU/L)**	1	128.3±4	124.6±4.1	135±10.5
	15	142.3±15.2	177±4.1	208.6±3.1
	30	165±4.7	256.3±15.3	308.6±8.8
**ALP(IU/L)**	1	443±30.4	390.6±20.9	459±21.9
	15	506±51.7	571±21.6	619.3±25.2
	30	508.6±14.5	625.3±13.3	884.6±24.8

## Discussion

### Growth during chronic cadmium exposure

Many studies have shown that the growth rate is lower in fish exposed to mixtures of metals through alterations of enzymatic capacity [30,31] and in some situations, alterations of the food basis in contaminated waters [32,33].

The reduction of fish growth by exposure to Cd of the current study may be due to the toxicity of the metal and consequently increase to the metabolic demands. Such increased metabolic demands disturb normal growth processes [34]. Waiwood and Beamish [35] presented evidence to suggest that exposure to Cd influences the basal metabolic rate of salmonids, which could limit growth through decreased efficiency of energy utilization coupled with increased metabolic maintenance costs. Qualitative observations of fish feeding behavior in the current study indicated that fish in Cd exposed groups did not fed actively and a severe reduction in feeding activity level and loss of appetite was observed in the high Cd exposure. The growth inhibition in the group receiving the highest cadmium dose, observed in our experiment, could be due to the influence of cadmium on food intake and assimilation because cadmium may cause disturbances in metabolic functions. Furthermore, it has been shown that cadmium decreases food intake and assimilation and this may lead to the reduction in the growth rate in fish [36]. In fact, the negative influence on growth is a well-known effect of cadmium action in fish and other aquatic organisms [28,37,38].

### Serum enzymes and parameters

Significant responses in the biochemical parameters generally followed by a similar pattern: an early elevation or depression followed a return to baseline values in chronic exposures to cadmium [39]. This pattern is suggestive of acclimation to the toxicant over time. The heavy metal may cause injury to the organisms and the damaged tissues subsequently malfunction [40]. Thus it is suggested that enzyme bioassay can be used as a valued tool to assess a change or damage caused to an organism due to administration of heavy metals [41]. In the present study, the level of both AST and ALT increased linearly over a 30-day period and the higher concentration caused a more significant effect on fish. Similarly, continuous exposure to sub-lethal cadmium concentrations resulted in significantly elevated levels of both AST and ALT activity in *Oreochromis niloticus* exposed to 0.05 mg/l Cd of 20-days [42]. The highest increase in ALT was observed in 5-day exposed fish. Transaminases like ALT and AST play significant role in amino acid and protein metabolism and they may release into the plasma following tissue damage and dysfunction. El-Naga et al. [43] showed that plasma enzymes (AST and ALT) were greatly affected by exposure to Cd in marine fish *Mugil seheli* and after a transient reduction dring the first 2-days, activity of enzymes increased to reach levels similar to the control value. Similarly, Thirumavalavan [40] showed that the levels of AST and ALT activity increased in the tissues of *Oreochromis mossambicus* exposed to cadmium chloride due to necrosis and increases in the permeability of cell membrane resulting in the damage of tissues after 7 and 14 days.

Different factors such as life history, water quality, and exposure duration and cadmium concentration influence ALP activity. The present study showed a linear pattern of increasing ALP over time with Cd exposure resulting in recognizable physiological and functional alterations after 30-days. In contrast, *Oreochromis niloticus* exposed to 0.05 mg/l Cd during 30-days showed reduction in ALP activity [42]. The decrease in ALP activity might be a result of disturbance of the membrane transport system, although the increase in the activity may be related to tissue damage [44].

Changes of blood glucose are a good indicator of metal stress in fish [45] and alterations in the glucose level might be related to renal injury, liver damage, and lack of nutrition [46]. This study showed a dose- dependent increase of glucose level after first 15-days. Similarly, serum glucose levels of *C. carpio* exposed to sublethal concentrations of Cd for 10 days increased with increasing concentrations of Cd in the water [47]. Nevertheless, the level of glucose reduced for the next 30 days of the current study. The higher level of glucose established on the first days of exposure, might be a result of glycogenolysis (a release of glucose into blood from energy resources stored as glycogen in muscles and liver), initiated by hormones (cortisol and catecholamines) when organism was in unfavorable condition. The reduced level of glucose established at the end of exposure probably reflected the exhaustion of the energy reserves of the organism and an impaired capacity of fish to restore them and acclimatized condition [48].

Triglyceride functions primarily in providing cellular energy and can be used as an indicator of nutritional status. The present study showed a reduction in serum triglyceride concentration in 15 days and returned to control level after 30 days due to acclimation to the toxicant over time. Serum triglyceride concentration in *Oreochromis niloticus* exposed to 0.05 mg/l Cd during 30 day did not change when compared to control value [42]. Variations in serum triglyceride concentrations might be due to differences in exposure concentration, lipid metabolism, and glycogen storage impairment in different fish species.

Cholesterol concentration in the serum of cadmium exposed fish also showed a different pattern. The present study showed a reduction in cholesterol within 15-days, possibly due to tissue damage in the kidney. On the contrary, in *Oreochromis niloticus*, an increase in cholesterol was seen during a 21 day period due to cadmium [42]. This alteration in cholesterol concentration could be due to hazardous effects of metals on cell membrane. Thus, increase in cholesterol levels are good indicators of environmental stress in fishes.

Total protein (TP) measurements from previous studies also exhibited no consistent pattern of response; protein levels decreased or were unaffected by cadmium exposures. The present study showed an increase in a 30 day period of time. In contrast, when *Oreochromis niloticus* was exposed to 0.05 mg/l Cd during 30 day, no significant alteration occurred in protein concentration [42]. Changes in serum TP, may be due to liver damage, reduction absorption and protein loss and thus may be a good indicator of health status of fish.

## Conclusions

Growth and serum biochemical parameters could sensitively reflect environmental metal stress concerning the effects of metal contamination and fish health.

## Competing interests

The authors declare that they have no competing interests.

## Authors’ contributions

MSH (IST author) carried out the main studies, participated in the experimental design and drafted the manuscript. MKH (2nd author), performed the main experiment. AN (3rd author) cooperated in running the experiment. All authors read and approved the final manuscript.
